# Supplementary material for: A guanidine-based coronavirus replication inhibitor which targets the nsp15 endoribonuclease and selects for interferon-susceptible mutant viruses
Source: PLoS Pathog. 2025 Feb 11;21(2):e1012571. doi: 10.1371/journal.ppat.1012571 (PMC11856660; doi:10.1371/journal.ppat.1012571)
Supplement: S1 Appendix — (PDF) [file ppat.1012571.s001.pdf]

S1 Appendix: Chemistry

Table of contents

General methods	S1
Chemical synthesis of **EPB-102**	S2
Chemical synthesis of **EPB-113**	S3
Chemical synthesis of **JMLv-061**	S6
^{1}H, ^{13}C and ^{19}F NMR spectra of **EPB-102**	S8
^{1}H, ^{13}C and ^{19}F NMR spectra of **EPB-113**	S10
^{1}H and ^{13}C spectra of **JMLV-061**	S12
References	S13

Chemical synthesis. General methods.

Commercially available reagents and solvents were used without further purification unless stated otherwise. Preparative normal phase chromatography was performed on a CombiFlash Rf 150 (Teledyne Isco) with pre-packed RediSep Rf silica gel cartridges. Thin-layer chromatography was performed with aluminum-backed sheets with silica gel 60 F_{254} (Merck, ref 1.05554), and spots were visualized with UV light and 1% aqueous solution of $KMnO_4$. Melting points were determined in open capillary tubes with a MFB 595010M Gallenkamp. 400 MHz ^{1}H, 100.6 MHz ^{13}C and 376.5 MHz ^{19}F NMR spectra were recorded on a Varian Mercury 400 or on a Bruker 400 Avance III spectrometers. The chemical shifts are reported in ppm (δ scale) relative to internal tetramethylsilane, and coupling constants are reported in Hertz (Hz). Assignments given for the NMR spectra of the new compounds have been carried out on the basis of COSY ^{1}H/^{13}C (gHSQC sequence) experiments. IR spectra were run on a Perkin-Elmer Spectrum RX I spectrophotometer. Absorption values are expressed as wave-numbers (cm^{-1}); only significant absorption bands are given. High-resolution mass spectrometry (HRMS) analyses were performed with an LC/MSD TOF Agilent Technologies spectrometer. The elemental analyses were carried out in a Flash 1112 series Thermofinnigan elemental microanalyzator (A5) to determine C, H, N and S. The structure of all new compounds was confirmed by elemental analysis and/or accurate mass measurement, IR, ^{1}H NMR, ^{13}C NMR and ^{19}F NMR. The analytical samples of all the new compounds, which were subjected to antiviral evaluation, possessed purity ≥ 95% as evidenced by their elemental analyses and/or their HPLC/MS. HPLC/MS for EPB-113 was determined with a HPLC Agilent 1260 Infinity II LC/MSD coupled to a photodiode array and mass spectrometer. 5 µL of sample 0.5 mg/mL in methanol:acetonitrile were injected, using a Agilent Poroshell 120 EC-C18, 2.7 µm, 50 mm x 4.6 mm column at 40 °C. The mobile phase was a mixture of A = water with 0.05% formic acid and B = acetonitrile with 0.05% formic acid, with the method described as follows: flow 0.6 mL/min, from 95% A - 5% B to 100% B in 3 min, 100% B 3 min, from 100% B to 95% A - 5% B in 1 min, 95% A – 5% B 3 min. Purity is given as % of absorbance at 254 nm. Data from mass spectra were analyzed by electrospray ionization in positive and negative between 100 and 1000 Da.

Synthesis of 1-(3-aminophenyl)-3-(3-(pentafluoro-λ^6-sulfanyl)phenyl)urea, EPB-102

Step 1: Synthesis of 1-(3-nitrophenyl)-3-(3-(pentafluoro-λ^6-sulfanyl)phenyl)urea

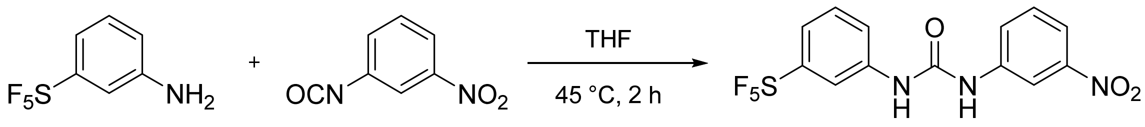

3-Nitrophenylisocyanate (500 mg, 3.05 mmol) was dissolved in THF (25 mL) and 3-pentafluorosulfanyl aniline (668 mg, 3.05 mmol) was added. The reaction mixture was stirred at 45 °C for 2 hours. The solvent was removed under reduced pressure to afford a yellow solid (1.10 g) that was crystallized in EtOAc to give pure 1-(3-nitrophenyl)-3-(3-(pentafluoro-λ^6-sulfanyl)phenyl)urea (1.10 g, 94% yield) as a yellow crystalline solid.

Step 2: Synthesis of EPB-102

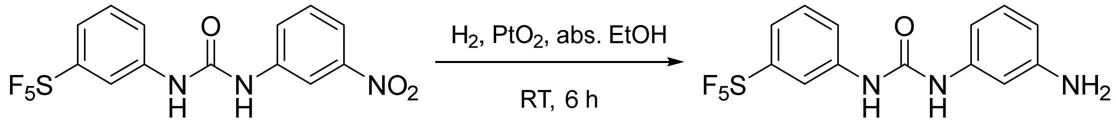

A suspension of 1-(3-nitrophenyl)-3-(3-(pentafluoro-λ^6-sulfanyl)phenyl)urea (600 mg, 1.56 mmol) and PtO_2 hydrate (53 mg) in abs. ethanol (45 mL) was hydrogenated (1 atm) at room temperature for 6 hours. The suspension was then filtered, and the solvent was evaporated under vacuum to afford **EPB-102** (533 mg, 97% yield) as a white solid. The analytical sample was obtained by crystallization from EtOAc, mp (EtOAc) 193–194 °C. IR (ATR) ν: 643, 651, 682, 697, 789, 824, 845, 877, 913, 1167, 1229, 1313, 1420, 1465, 1485, 1566, 1590, 1651, 3092, 3294, 3389, 3489 cm^{-1}. ^{1}H-NMR (400 MHz, DMSO-d_6) δ: 5.05 (s, 2 H, NH_2), 6.21 (m, 1 H, 6-H or 4-H), 6.52 (m, 1 H, 4-H or 6-H), 6.84 (t, J = 2.0 Hz, 1 H, 2-H), 6.90 (t, J = 8.0 Hz, 1 H, 5-H), 7.40-7.55 (complex signal, 3 H, 4'-H, 5'-H, 6'-H), 8.28 (t, J = 2.0 Hz, 1 H, 2'-H), 8.51 (s, 1 H) and 9.03 (s, 1 H) (2 NH). ^{13}C-NMR (100.6 MHz, DMSO-d_6) δ: 104.1 (CH, C2), 106.3 (CH, C4 or C6), 108.4 (CH, C6 or C4), 114.6 (quint, $^3J_{CF}$ = 5.0 Hz, CH, C2'), 118.5 (quint, $^3J_{CF}$ = 3.5 Hz, CH, C4'), 121.5 (CH, C6'), 129.0 (CH, C5), 129.6 (CH, C5'), 139.8 (C, C1), 140.7 (C, C1'), 149.2 (C, C3), 152.3 (C, CO), 153.3 (quint, $^2J_{CF}$ = 15.1 Hz, C, C3'). ^{19}F-NMR (376.5 MHz, DMSO-d_6) δ: 63.6 (d, J = 150.6 Hz, 4 F, $S\underline{F}_4F$), 87.8 (quint, J = 150.6 Hz, 1 F, $SF_4\underline{F}$). HRMS-ESI$^-$ m/z [M-H]$^-$ calculated for $[C_{13}H_{12}F_5N_3OS\text{-}H]^-$: 352.0548. Found: 352.0554. Elemental analysis calculated for $C_{13}H_{12}F_5N_3OS$: C 44.19%, H 3.42%, N 11.89%, S 9.07%. Found: C 44.24%, H 3.44%, N 11.63%, S 8.78%.

Synthesis of 1-(3-guanidinophenyl)-3-(3-(pentafluoro-λ^6-sulfanyl)-phenyl)urea hydrochloride, EPB-113

Step 1: Synthesis of 2-(3-nitrophenyl)-1,3-di-Boc-guanidine

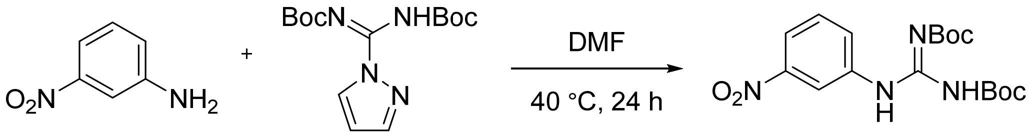

To a stirred solution of 3-nitroaniline (1 g, 7.24 mmol) in DMF (10 mL) was added bis-Boc-pyrazolocarboxamidine (2.25 g, 7.25 mmol) and the reaction mixture was stirred at 40 °C for 24 hours. Water (15 mL) and EtOAc (15 mL) were added, and the phases were separated. The organic layer was further washed with water, dried over anhydrous Na_2SO_4 and filtered. Evaporation of the organics provided a yellowish gum (2.46 g), which was purified by column chromatography in silica gel (hexane/EtOAc mixtures). Fractions containing the desired product were collected and concentrated under vacuum to afford 2-(3-nitrophenyl)-1,3-di-Boc-guanidine (1.0 g, 36% yield) as a yellowish solid. The spectroscopic data matched with those previously described in the bibliography.[1] HRMS-ESI$^+$ *m/z* $[M+H]^+$ calculated for $[C_{17}H_{24}N_4O_6+H]^+$: 381.1769. Found: 381.1769.

Step 2: Obtention of 2-(3-aminophenyl)-1,3-di-Boc-guanidine

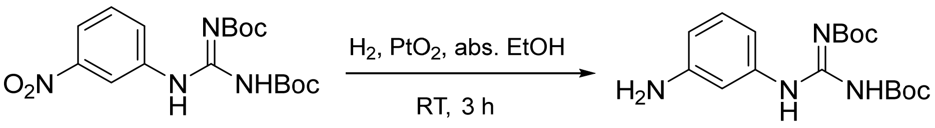

A suspension of 2-(3-nitrophenyl)-1,3-di-Boc-guanidine (1 g, 2.63 mmol) and PtO_2 hydrate (100 mg) in abs. ethanol (100 mL) was hydrogenated (1 atm) at room temperature for 3 hours. The suspension was then filtered, and the solvent was evaporated under vacuum to afford 2-(3-aminophenyl)-1,3-di-Boc-guanidine (0.91 g, quantitative yield) as a white foamy solid. The spectroscopic data matched with those previously described in the bibliography.[2]

Step 3: Synthesis of 1-(1,3-di-Boc-3-guanidinophenyl)-3-(3-(pentafluoro-λ^6-sulfanyl)phenyl)urea

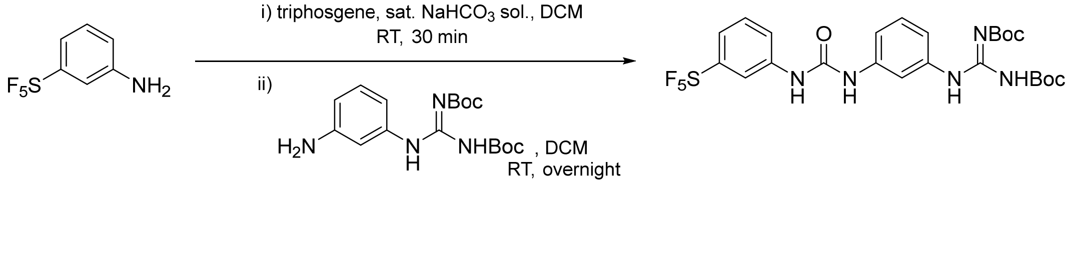

i) 3-pentafluorosulfanyl aniline (80 mg, 0.36 mmol) was added to a stirring biphasic mixture of DCM (5 mL) and saturated aqueous $NaHCO_3$ solution (5 mL). Triphosgene (40 mg, 0.14 mmol) was then slowly added and the reaction mixture was stirred at room temperature for 30 minutes. The phases were separated and the organic layer was washed with brine, dried over anhydrous Na_2SO_4, filtered and half concentrated at room temperature to give 3-(pentafluoro-λ^6-sulfanyl)phenylisocyanate in DCM solution that was used in the next step without further purification.

ii) 2-(3-aminophenyl)-1,3-di-Boc-guanidine (139 mg, 0.40 mmol) dissolved in DCM (2 mL) was added to the previously obtained isocyanate in DCM (ca. 6 mL). The reaction mixture was stirred at room temperature overnight. Evaporation under vacuum of the solvent provided an orange solid (350 mg) which was purified by column chromatography in silica gel (hexane/EtOAc mixtures). Fractions containing the desired product were collected and concentrated under vacuum to give 1-(1,3-di-Boc-3-guanidinophenyl)-3-(3-(pentafluoro-λ^6-sulfanyl)phenyl)urea (60 mg, 29% overall yield) as a yellowish solid. The analytical sample was obtained by washing with Et_2O. ^{1}H-NMR (400 MHz, DMSO-d_6) δ: 1.41 (s, 9 H, Boc), 1.51 (s, 9 H, Boc), 7.15-7.35 (complex signal, 3 H, 2-H, 4-H, 6-H), 7.41-7.59 (complex signal, 3 H, 4'-H, 5-H, 6'-H), 7.64 (s, 1 H, 5'-H), 8.27 (m, 1 H, 2'-H), 8.93 (s, 1 H) and 9.17 (s, 1 H) (2 NH urea), 9.98 (s, 1 H) and 11.35 (s, 1 H) (2 NH guanidine).

Step 4: Synthesis of EPB-113 (as its hydrochloride salt)

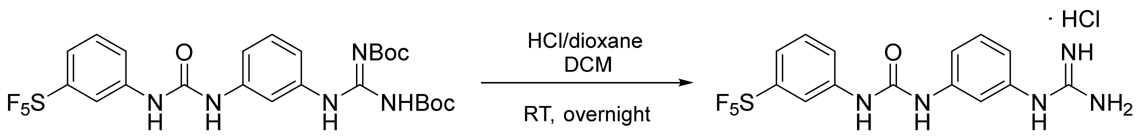

To a stirred suspension of 1-(1,3-di-Boc-3-guanidinophenyl)-3-(3-(pentafluoro-λ^6-sulfanyl)phenyl)urea (32 mg, 0.05 mmol) in DCM (1 mL) was added a 4 M solution of HCl in 1,4-dioxane (4 mL, 16 mmol) and the mixture was stirred at room temperature overnight. Evaporation of the solvents provided a beige solid, which was washed with Et_2O and pentane to give **EPB-113** as its hydrochloride salt (19 mg, 88% yield), mp 219–220 °C. IR (ATR) ν: 754, 782, 812, 835, 857, 916, 1098, 1239, 1296, 1406, 1428, 1484, 1496, 1542, 1587, 1598, 1673, 2966, 3184, 3235, 3416 cm^{-1}. ^{1}H-NMR (400 MHz, DMSO-d_6) δ: 6.86 (m, 1 H, 4-H), 7.27 (m, 1 H, 6-H), 7.35 (t, J = 8.0 Hz, 1 H, 5-H), 7.38 (broad s, 4 H, 2 $N\underline{H}_2$ guanidine), 7.46-7.55 (complex signal, 4 H, 2-H, 4'-H, 5'-H, 6'-H), 8.27 (m, 1 H, 2'-H), 9.31 (s, 1 H) and 9.53 (s, 1 H) (2 NH urea), 9.70 (s, 1 H, 3-NH). ^{13}C-NMR (100.6 MHz, CD_3OD) δ: 117.2 (CH, C2), 117.4 (quint, $^3J_{CF}$ = 5.0 Hz, CH, C2'), 119.4 (CH, C6), 120.5 (CH, C4), 120.9 (quint, $^3J_{CF}$ = 5.0 Hz, CH, C4'), 123.1 (CH, C6'), 130.3 (CH, C5'), 131.4 (CH, C5), 136.6 (C, C1), 141.3 (C, C1'), 142.0 (C, C3), 154.8 (C, CO), 155.4 (quint, $^2J_{CF}$ = 17.1 Hz, C, C3'), 158.0 (C, CN guanidine). ^{19}F-NMR (376.5 MHz, DMSO-d_6) δ: 63.6 (d, J = 150.6 Hz, 4 F, $S\underline{F}_4F$), 87.7 (quint, J = 150.6 Hz, 1 F, $SF_4\underline{F}$). HRMS-ESI$^+$ m/z $[M+H]^+$ calculated for $[C_{14}H_{14}F_5N_5OS+H]^+$: 396.0912. Found: 396.0916. HPLC (254 nm): t_R = 3.75 min (98.4%).

Elemental analysis calculated for $C_{14}H_{14}F_5N_5OS \cdot HCl \cdot 0.4H_2O$: C 38.30%, H 3.63%, N 15.95%. Found: C 38.51%, H 3.88%, N 16.21%.

Synthesis of 1-(3-guanidinophenyl)-3-(2-(trifluoromethoxy)phenyl)urea dihydrochloride, JMLV-061

Step 1: Synthesis of 2-(3-nitrophenyl)-1,3-di-Boc-guanidine

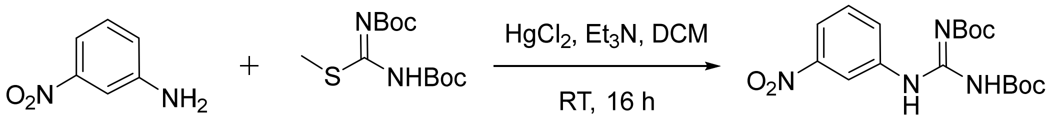

To a solution of 3-nitroaniline (1.50 g, 10.86 mmol) and triethylamine (4.54 mL, 32.58 mmol) in anhydrous DCM (40 mL) was added *N,N'*-bis(tert-butoxycarbonyl)-S-methylisothiourea (3.31 g, 11.40 mmol) and $HgCl_2$ (3.24 g, 11.95 mmol) and the mixture was stirred at RT for 16 hours. The mixture was filtered through a pad of Celite® using DCM as eluting agent. Then, the solution was washed with water (2 x 80 mL). The organic layer was dried over anhydrous Na_2SO_4 and filtered. Solvents were concentrated under vacuum and the resulting crude was purified by column chromatography in silica gel (hexane/EtOAc mixtures). Fractions containing the desired product were collected and concentrated under vacuum to afford 2-(3-nitrophenyl)-1,3-di-Boc-guanidine as a pale-yellow solid (3.88 g, 94% yield). The spectroscopic data matched with those previously described in the bibliography.[1] HRMS-ESI$^+$ *m/z* $[M+H]^+$ calculated for $[C_{17}H_{24}N_4O_6+H]^+$: 381.1769. Found: 381.1769.

Step 2: Obtention of 2-(3-aminophenyl)-1,3-di-Boc-guanidine

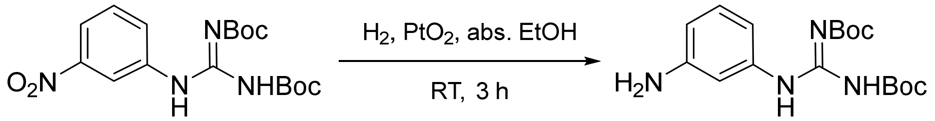

A suspension of 2-(3-nitrophenyl)-1,3-di-Boc-guanidine (2.28 g, 5.99 mmol) and PtO_2 hydrate (250 mg) in abs. ethanol (50 mL) was hydrogenated at room temperature and atmospheric pressure for 3 hours. Anhydrous Na_2SO_4 and Celite® were added and the mixture was stirred for 2 minutes. Then, the suspension was filtered and the solvent was evaporated under vacuum. The resulting crude was purified by column chromatography in silica gel (hexane/EtOAc mixtures). Fractions containing the desired product were collected and concentrated under vacuum to afford 2-(3-aminophenyl)-1,3-di-Boc-guanidine (1.75 g, 83% yield) as a white solid. The spectroscopic data matched with those previously described in the bibliography.[2]

Step 3: Synthesis of JMLV-061 (as its hydrochloride)

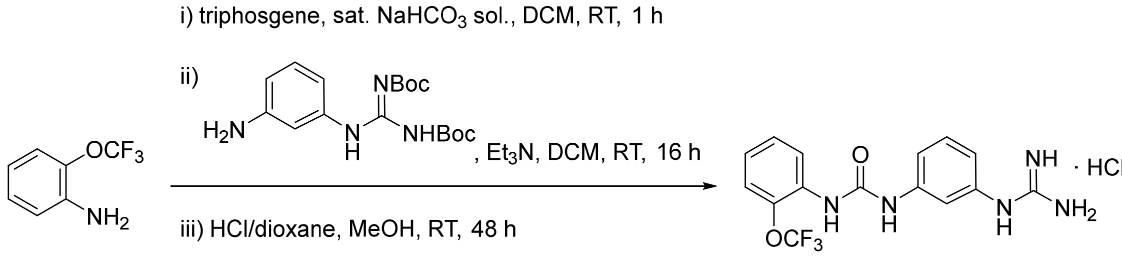

i) To a stirring biphasic mixture of DCM (3 mL), saturated aqueous $NaHCO_3$ solution (3 mL) and triphosgene (101 mg, 0.34 mmol) was added 2-(trifluoromethoxy)aniline (93 µL, 120 mg, 0.68 mmol) portionwise. The mixture was then stirred at room temperature for 1 hour. The phases were separated and the organic layer was washed with brine, dried over anhydrous Na_2SO_4 and filtered. Solvent was concentrated until 1 mL of DCM was left to give 2-(trifluoromethoxy)phenylisocyanate in solution that was used in the next step without further purification.

ii) 2-(3-aminophenyl)-1,3-di-Boc-guanidine (80 mg, 0.23 mmol) and triethylamine (128 µL, 93 mg, 0.92 mmol) dissolved in DCM (2 mL) was added to the previous isocyanate solution and the mixture was stirred at room temperature for 16 hours. Saturated aqueous $NaHCO_3$ solution (15 mL) followed by DCM (10 mL) were added to the mixture and layers were separated. The organic layer was washed again with saturated aqueous $NaHCO_3$ solution (15 mL). Then, the organic layer was separated, dried over anhydrous Na_2SO_4, filtered and solvents were concentrated under vacuum to afford 1-(1,3-di-Boc-3-guanidinophenyl)-3-(2-(trifluoromethoxy)phenyl)urea that was used in the next step without further purification.

iii) A 4 M solution of HCl/1,4-dioxane (2 mL) and MeOH (0.5 mL) were added to the previously obtained crude and the mixture was stirred at room temperature for 48 hours. The solvents were concentrated under vacuum and the resulting crude was purified by column chromatography in silica gel (DCM/MeOH mixtures). Fractions containing the desired product were collected and concentrated under vacuum to afford **JMLv-061** in its dihydrochloride salt as a beige solid (39 mg, 48% yield), mp 218–219 °C. IR (ATR) ν: 624, 691, 758, 871, 1006, 1059, 1180, 1215, 1314, 1406, 1454, 1490, 1540, 1598, 1674, 2421, 3200, 3330 cm^{-1}. ^{1}H-NMR (400 MHz, CD_3OD) δ: 6.95 (ddd, *J* = 8.0 Hz, *J'* = 2.1 Hz, *J''* = 1.0 Hz, 1 H, 4-H), 7.11 (ddd, *J* = 8.1 Hz, *J'* = 7.5 Hz, *J''* = 1.6 Hz, 1 H, 4'-H), 7.29 (m, 1 H, 6-H), 7.30-7.34 (complex signal, 2 H, 3'-H, 5'-H), 7.40 (t, *J* = 8.0 Hz, 1 H, 5-H), 7.64 (t, *J* = 2.1 Hz, 1 H, 2-H), 8.20 (dd, *J* = 8.8 Hz, *J'* = 1.6 Hz, 1 H, 6'-H). ^{13}C-NMR (100.6 MHz, CD_3OD) δ: 116.8 (CH, C2), 119.0 (CH, C6), 120.4 (CH, C4), 122.11 (q, $^{1}J_{CF}$ = 256.5 Hz, C, OCF_3), 122.13 (CH, C3'), 123.1 (CH, C6'), 124.4 (CH, C4'), 128.7 (CH, C5'), 131.5 (CH, C5), 133.1 (C, C1'), 136.7 (C, C1), 139.9 (C, C2'), 142.1 (C, C3), 154.6 (C, CO), 158.0 (C, CN guanidine). HRMS-ESI$^+$ *m/z* $[M+H]^+$ calculated for $[C_{15}H_{14}F_3N_5O_2+H]^+$: 354.1172. Found: 354.1174. Elemental analysis calculated for $C_{15}H_{14}F_3N_5O_2$·HCl·0.6DCM: C 42.51%, H 3.70%, N 15.89%. Found: C 42.38%, H 3.90%, N 15.70%.

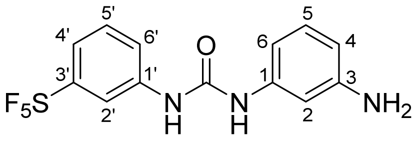
F5S
NH2
O
N
H
1
2
3
4
5
6
1'
2'
3'
4'
5'
6'

EPB-102

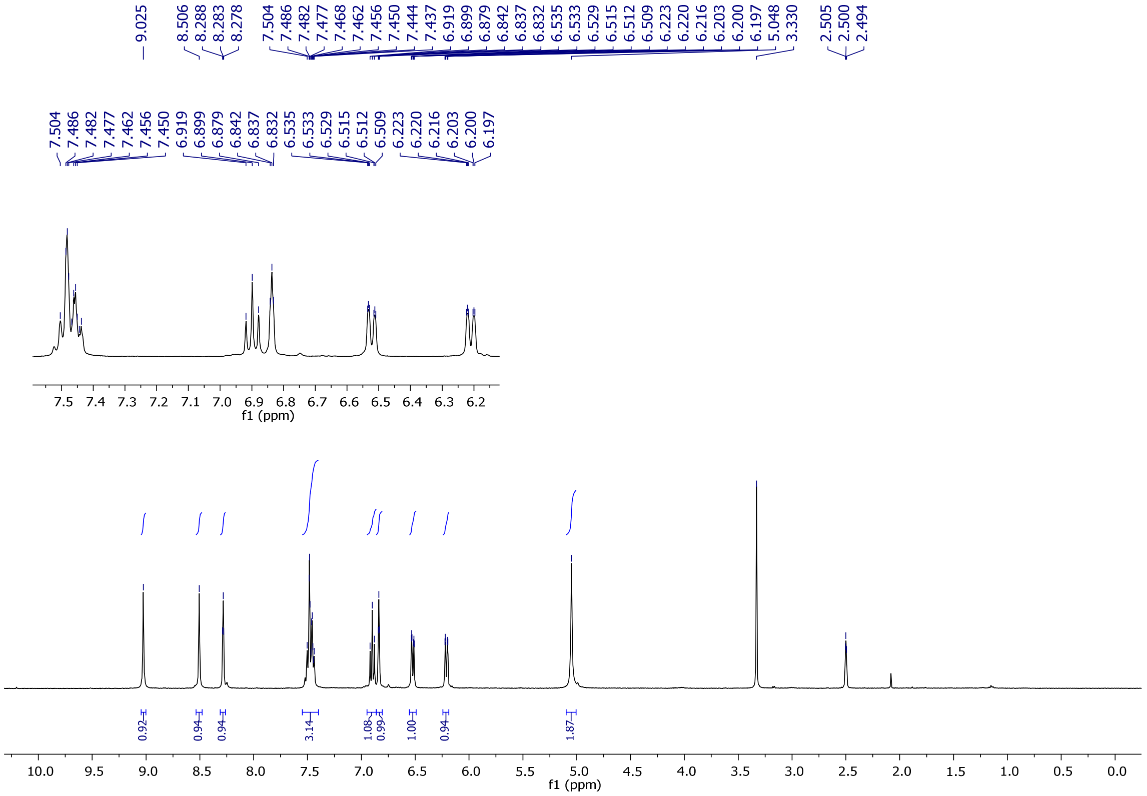
9.025
8.506
8.288
8.283
8.278
7.504
7.486
7.482
7.477
7.468
7.462
7.456
7.450
7.444
7.437
6.919
6.899
6.879
6.842
6.837
6.832
6.535
6.533
6.529
6.515
6.512
6.509
6.223
6.220
6.216
6.203
6.200
6.197
5.048
3.330
2.505
2.500
2.494
0.92
0.94
0.94
3.14
1.08
0.99
1.00
0.94
1.87
f1 (ppm)

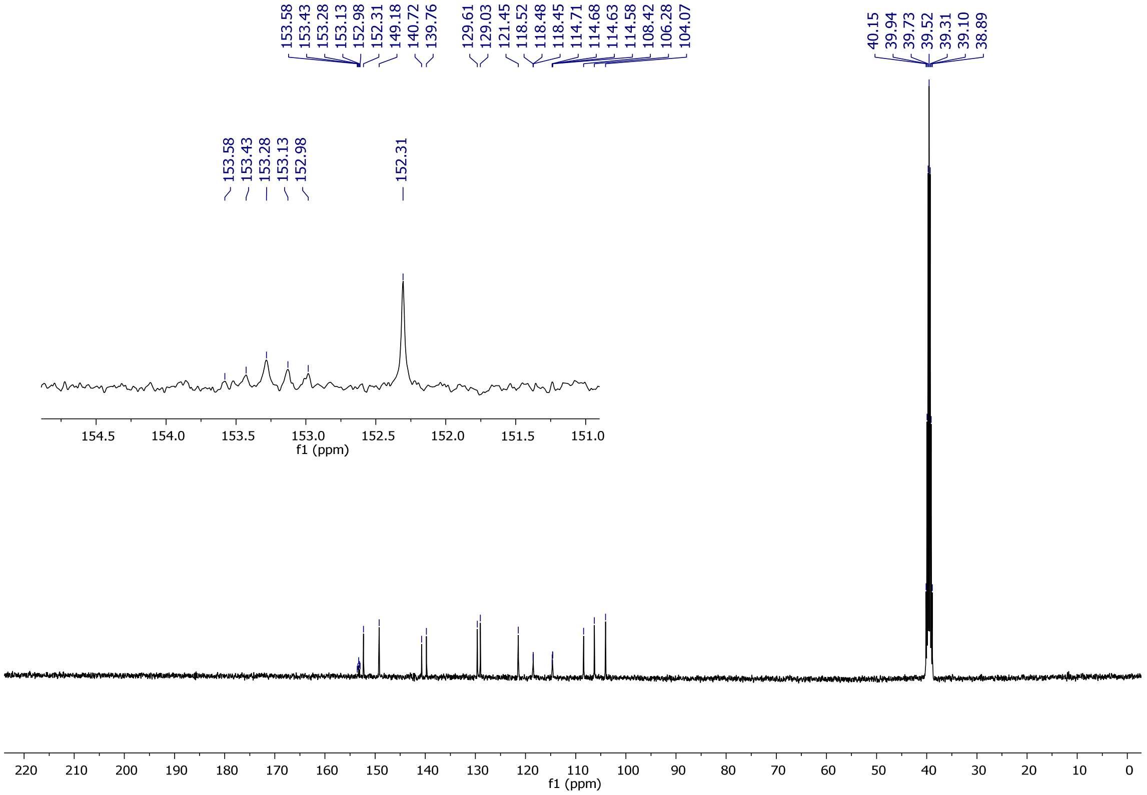
153.58
153.43
153.28
153.13
152.98
152.31
149.18
140.72
139.76
129.61
129.03
121.45
118.52
118.48
118.45
114.71
114.68
114.63
114.58
108.42
106.28
104.07
40.15
39.94
39.73
39.52
39.31
39.10
38.89
f1 (ppm)

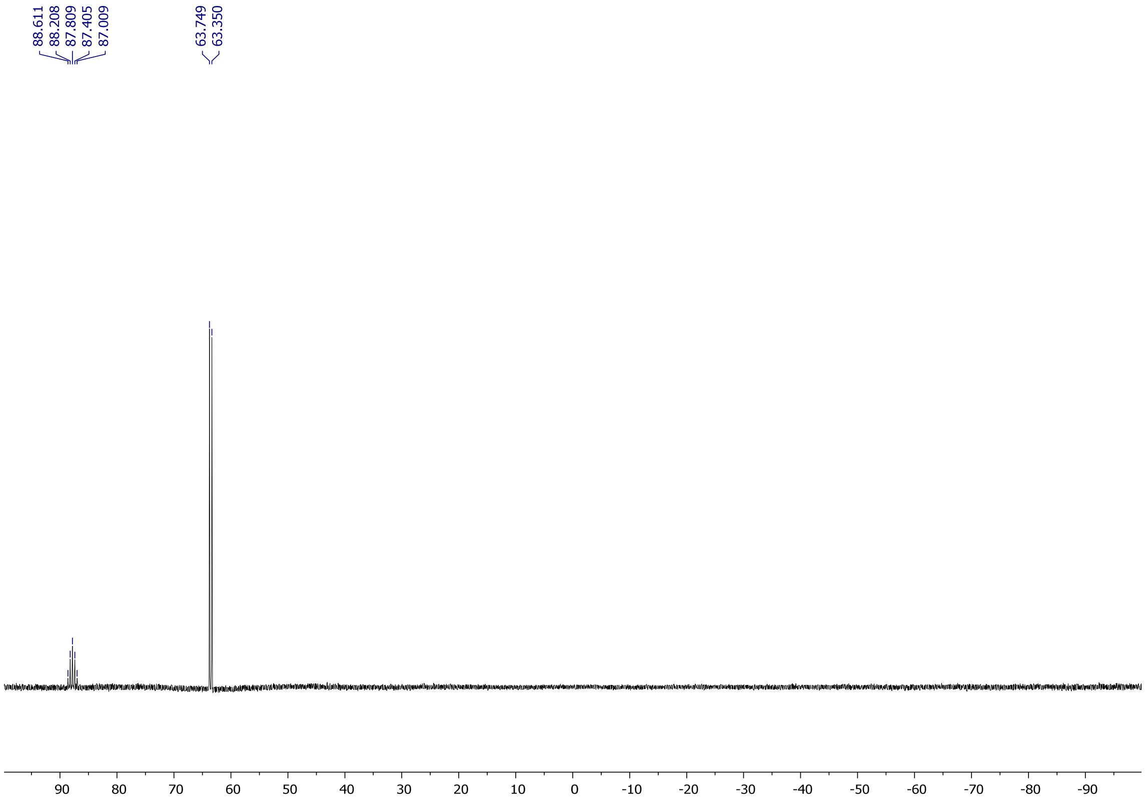
88.611
88.208
87.809
87.405
87.009
63.749
63.350

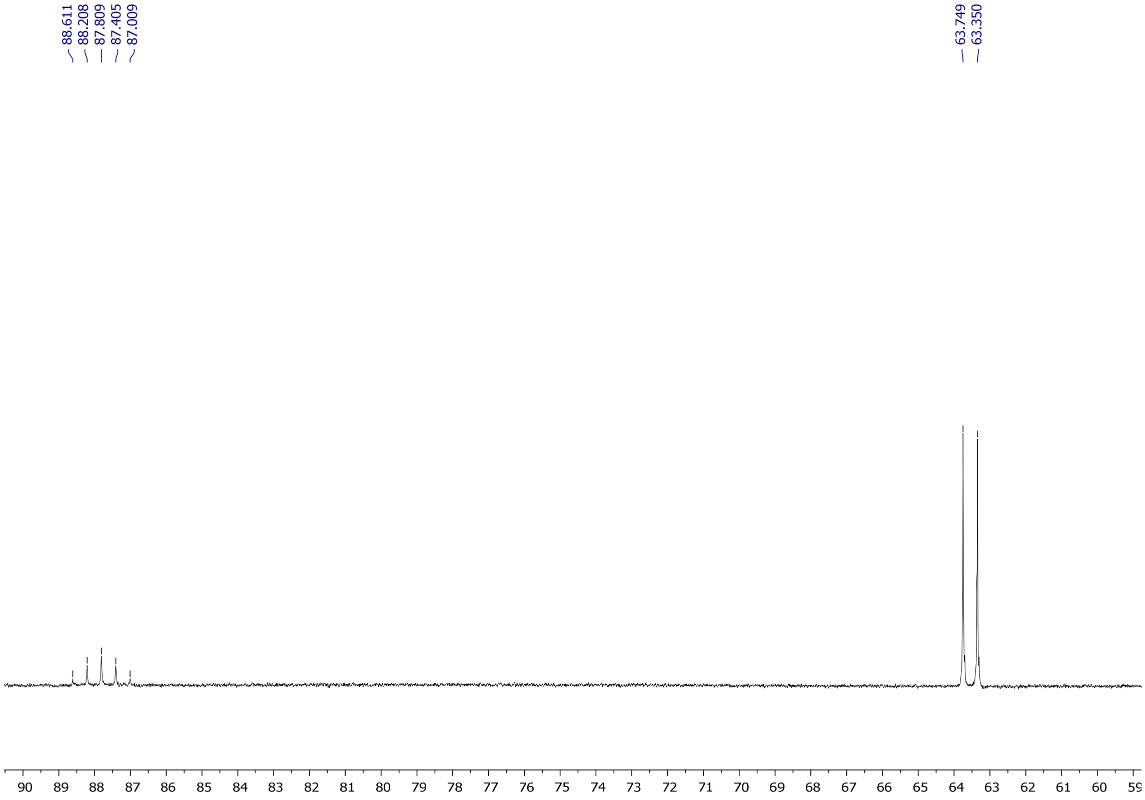
88.611
88.208
87.809
87.405
87.009
63.749
63.350

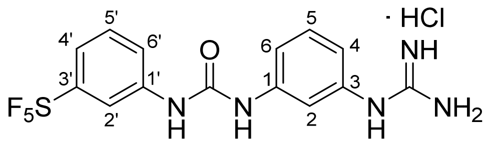
· HCl
NH
O
N
H
N
H
N
H
NH2
F5S
1'
2'
3'
4'
5'
6'
1
2
3
4
5
6

EPB-113

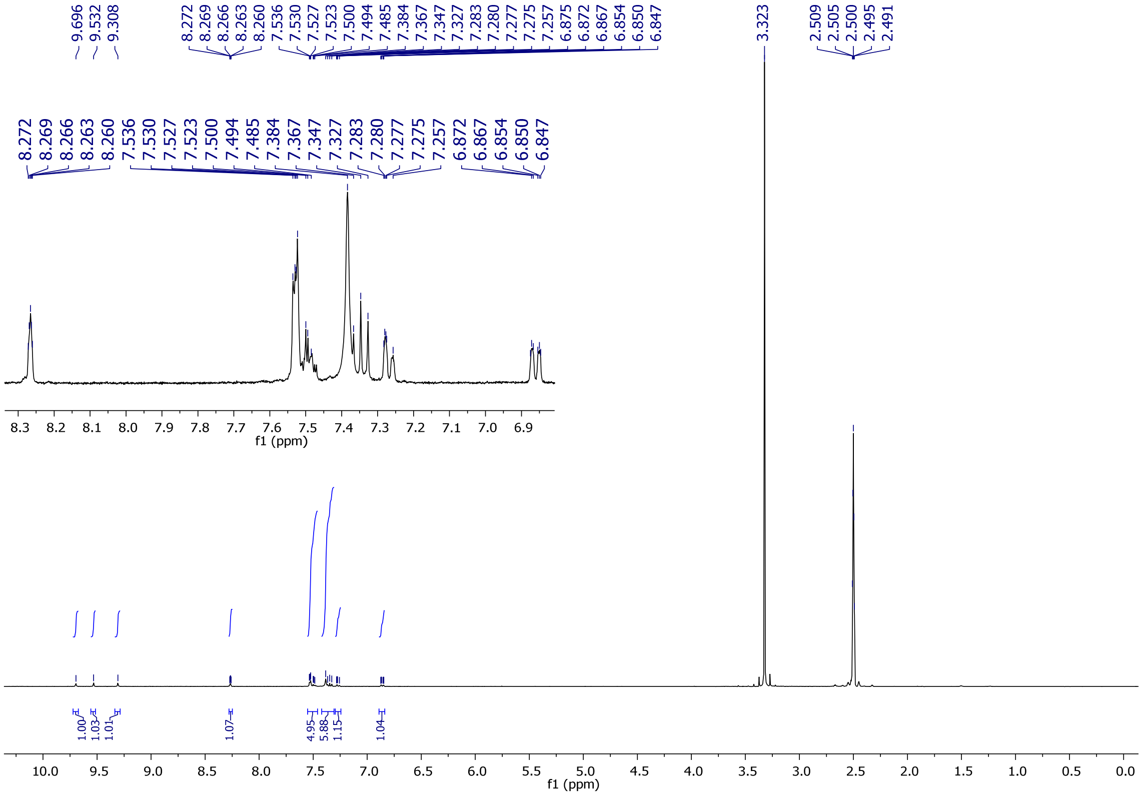
9.696
9.532
9.308
8.272
8.269
8.266
8.263
8.260
7.536
7.530
7.527
7.523
7.500
7.494
7.485
7.384
7.367
7.347
7.327
7.283
7.280
7.277
7.275
7.257
6.875
6.872
6.867
6.854
6.850
6.847
3.323
2.509
2.505
2.500
2.495
2.491
1.00
1.03
1.01
1.07
4.95
5.88
1.15
1.04
f1 (ppm)

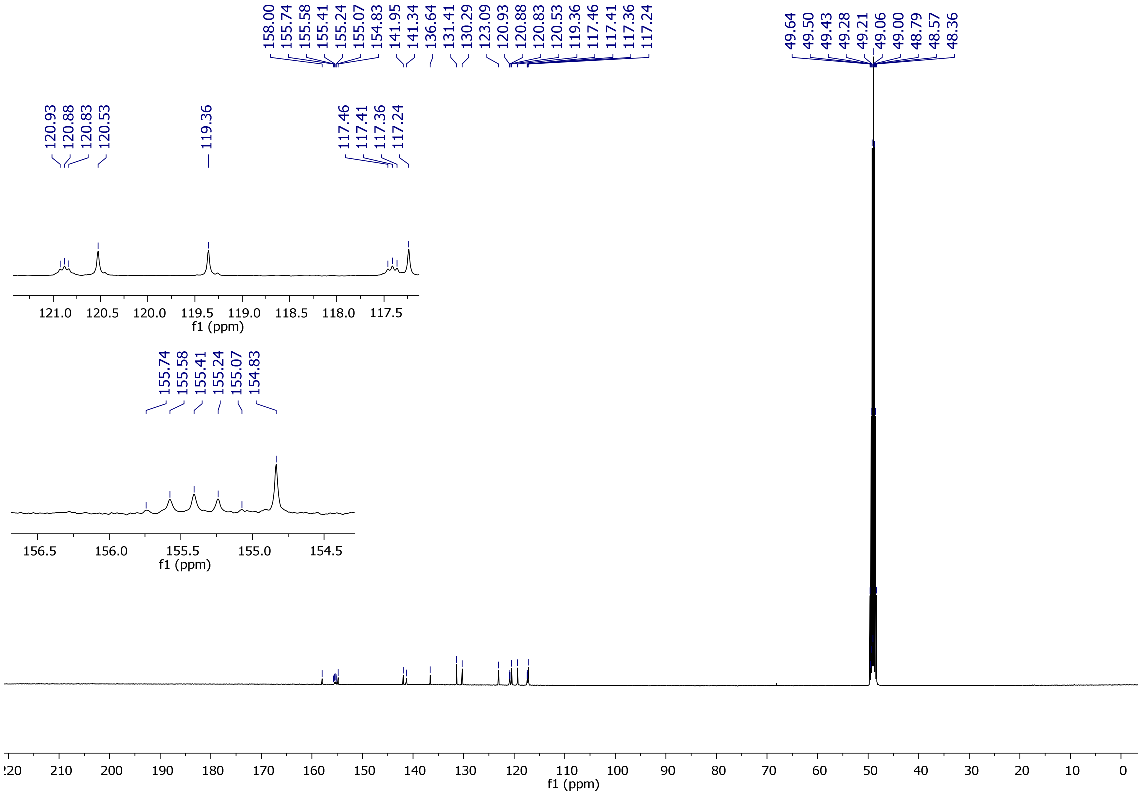
158.00
155.74
155.58
155.41
155.24
155.07
154.83
141.95
141.34
136.64
131.41
130.29
123.09
120.93
120.88
120.83
120.53
119.36
117.46
117.41
117.36
117.24
49.64
49.50
49.43
49.28
49.21
49.06
49.00
48.79
48.57
48.36
f1 (ppm)

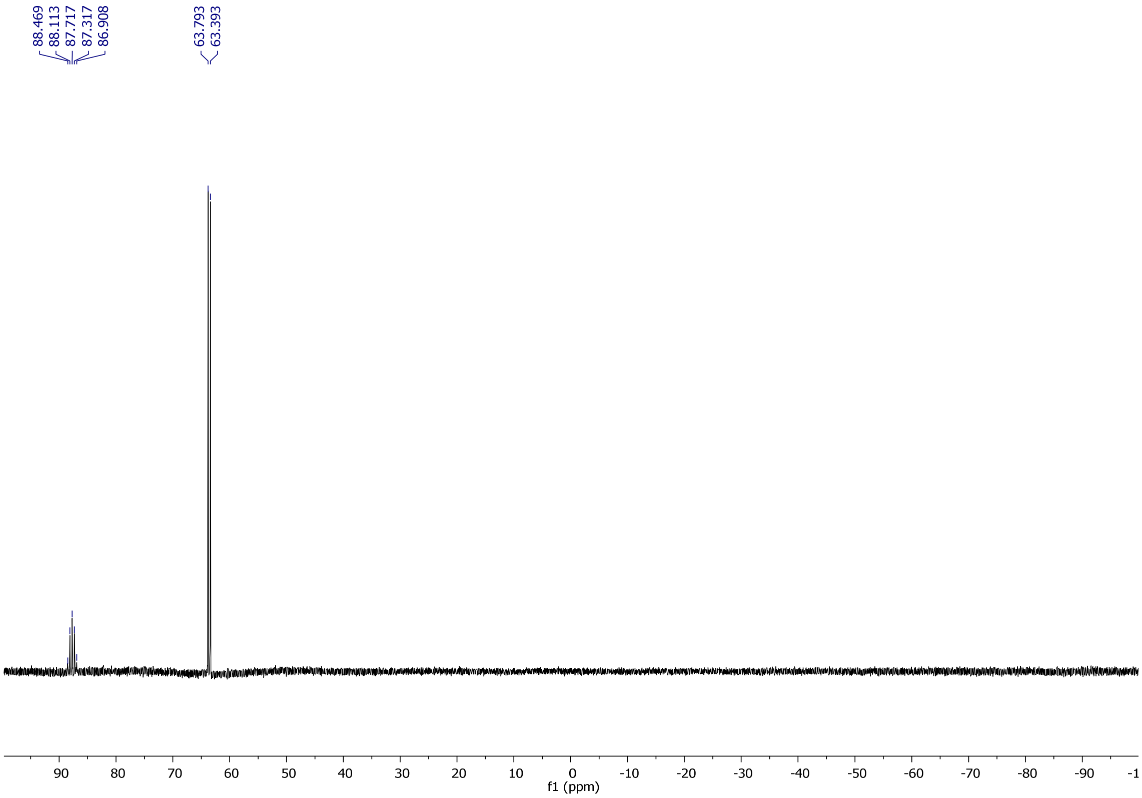
88.469
88.113
87.717
87.317
86.908
63.793
63.393
f1 (ppm)

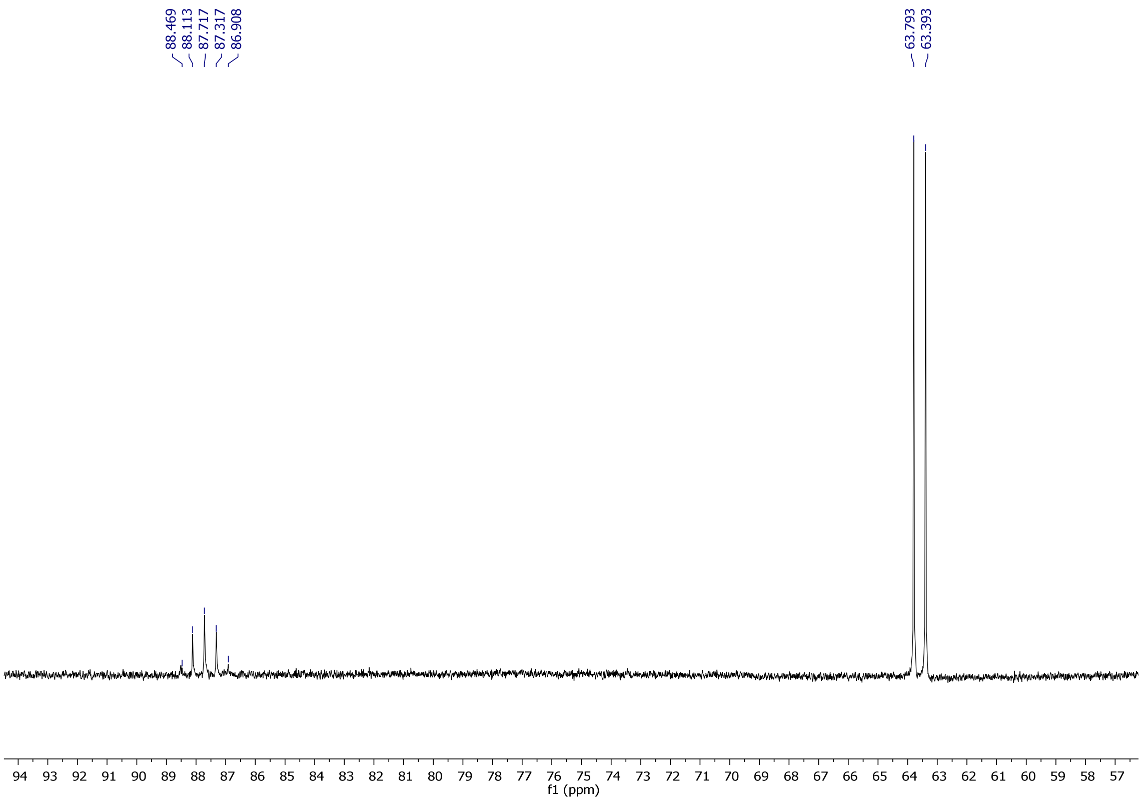
88.469
88.113
87.717
87.317
86.908
63.793
63.393
f1 (ppm)

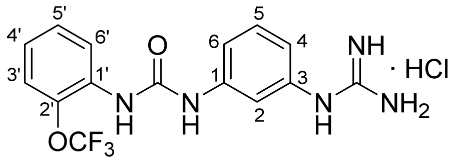
5'
4'
6'
3'
1'
2'
OCF3
O
N
H
N
H
6
5
4
1
3
2
NH
· HCl
N
H
NH2

JMLV-061

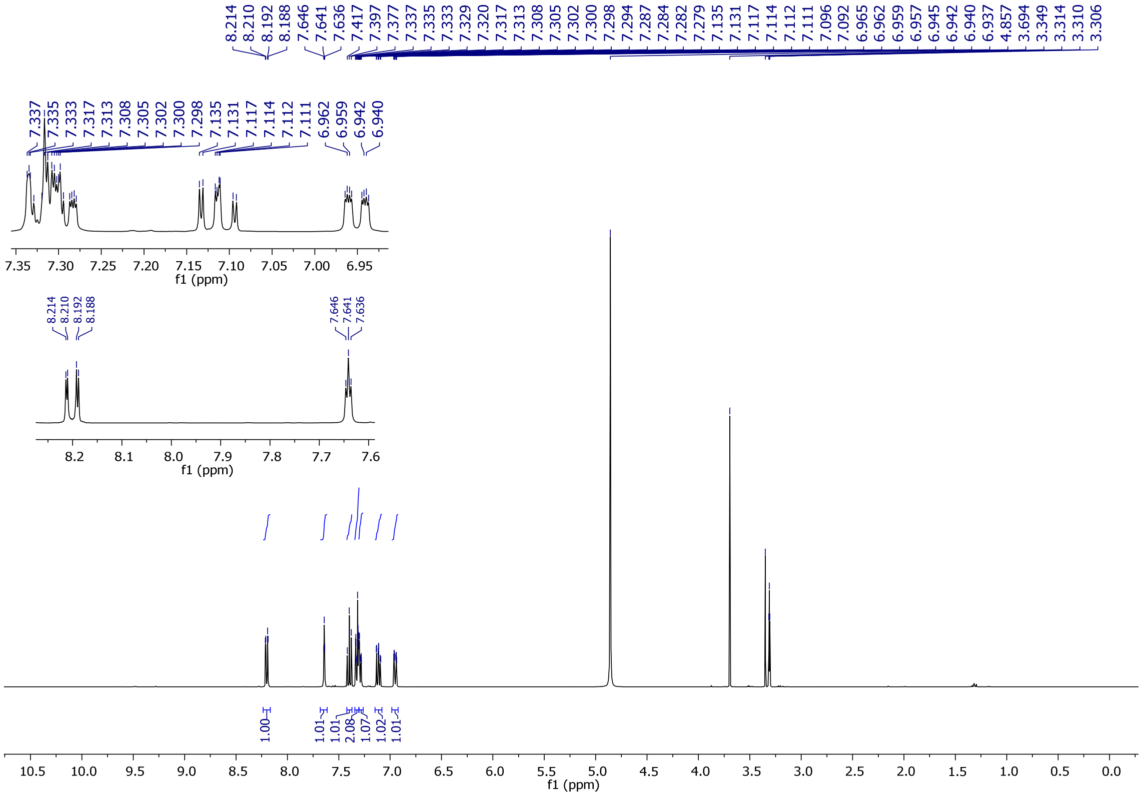
8.214
8.210
8.192
8.188
7.646
7.641
7.636
7.417
7.397
7.377
7.337
7.335
7.333
7.329
7.320
7.317
7.313
7.308
7.305
7.302
7.300
7.298
7.294
7.287
7.284
7.282
7.279
7.135
7.131
7.117
7.114
7.112
7.111
7.096
7.092
6.965
6.962
6.959
6.957
6.945
6.942
6.940
6.937
4.857
3.694
3.349
3.314
3.310
3.306
1.00
1.01
1.01
2.08
1.07
1.02
1.01
f1 (ppm)

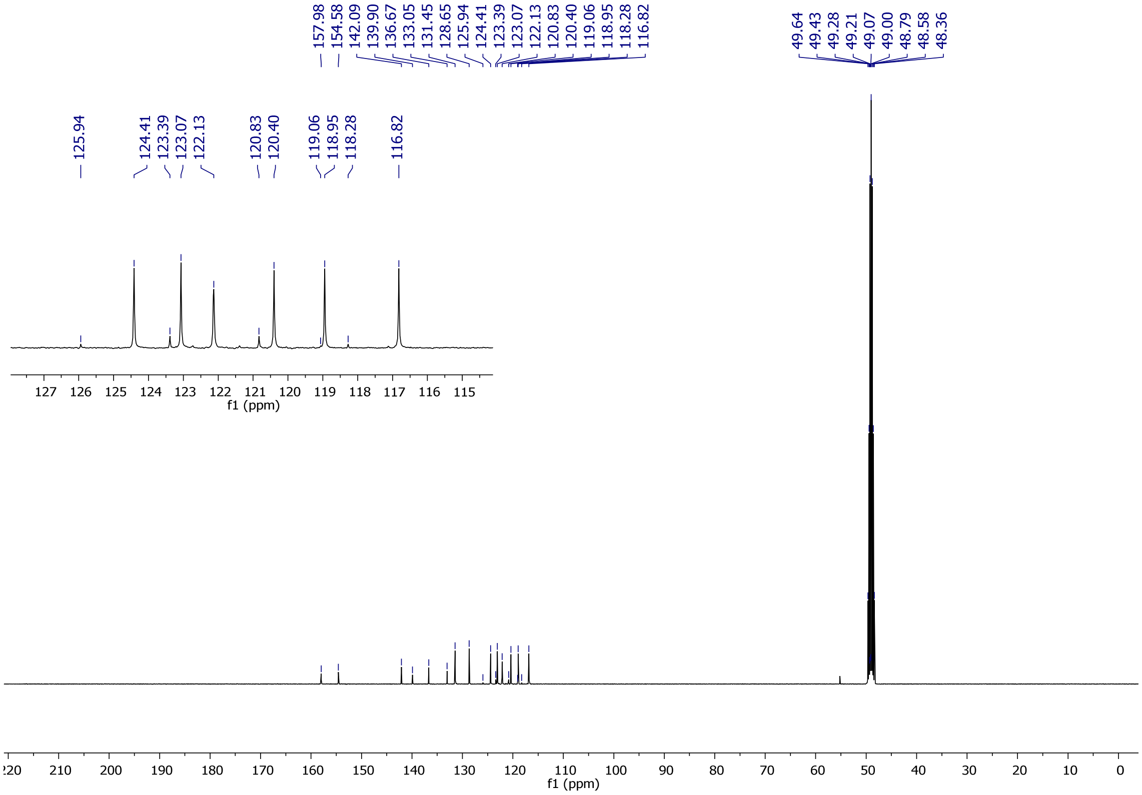
157.98
154.58
142.09
139.90
136.67
133.05
131.45
128.65
125.94
124.41
123.39
123.07
122.13
120.83
120.40
119.06
118.95
118.28
116.82
49.64
49.43
49.28
49.21
49.07
49.00
48.79
48.58
48.36
f1 (ppm)

References

1. Guo Z-X, Cammidge AN, Horwell DC. A convenient and versatile method for the synthesis of protected guanidines. Synt. Commun. 2000;30(16):2933-2943.

2. Kaloun EB, Schmitt P, Kruczynski, A. Isoquinolinone derivatives useful in the treatment of cancer. US9944641B2 (Patent) 2014.
