# Supplementary material for: A guanidine-based coronavirus replication inhibitor which targets the nsp15 endoribonuclease and selects for interferon-susceptible mutant viruses
Source: PLoS Pathog. 2025 Feb 11;21(2):e1012571. doi: 10.1371/journal.ppat.1012571 (PMC11856660; doi:10.1371/journal.ppat.1012571)
Supplement: S2 Appendix — Oligonucleotide sequences used for sequencing or (RT-)qPCR. (PDF) [file ppat.1012571.s002.pdf]

S2 Appendix: Primer and probe sequences

One-Step RT-PCR for sequencing of HCoV-229E: five primer pairs

Fragment 1	229E-For84	CTATGGCCGGCATCTTTGA	and	229E-Rev6270	CAAGCAGCCACCACTTAGAT
Fragment 2	229E-For5967	CAGTTGTTATGGTTGGTGGTTATG	and	229E-Rev12295	TATGTGTCCTGCGTAGTGTTG
Fragment 3	229E-For11960	GAACCACCTTGCAGATTTGTTATAG	and	229E-Rev18300	GACCCACCATTTACACCTTCA
Fragment 4	229E-For17743	GTTGCTTCTGGTGATGCTATTATG	and	229E-Rev24484	ACGTACAAGTCGTTAGTTGAGAG
Fragment 5	229E-For23936	TCAGTCGTGCTCATCTTTGTG	and	229E-Rev27281	TTTGTGTATCCATATCGAAACCGT

Sequencing of PCR fragments HCoV-229E

Fragment 1	229E-For84	CTATGGCCGGCATCTTTGA
	229E-Rev454	AACGATGCAATCCTGCAAATC
	229E-For854	CTACGCAATGGTTCTGTTCTTG
	229E-For1679	GACGTGTTTGTCACAGCTATTC
	229E-For2527	CACACAACAGGGTTTGCTTG
	229E-For3251	TCTGAATGGCCTCTTTCTGTTC
	229E-For4100	GAGAAGTTGAACGCCTTCCT
	229E-For4910	TGTGTTGTGCCATCCGAAA
	229E-For5709	ATGGTGTGCAAGTTGGTTATTG
Fragment 2	229E-For5967	CAGTTGTTATGGTTGGTGGTTATG
	229E-For6682	GATGAACTGCTTGTCACTGTTATT
	229E-For6757	CCCAGATTGTATTGCGTGTTC
	229E-For7617	CTGCTGAAGGTCGCAAGTAT
	229E-For8353	GCGTACTATTGCCACCAAATAC
	229E-For9213	CATCTGGCTTTGTGGAGAAATG
	229E-For9947	ACTGGTGTCTGTGTGGAAAG
	229E-For10793	TGTGTTAGTCCAGCCGAATTTA
	229E-For11627	GCTGGTGTTGTTTGGACATTAC
Fragment 3	229E-For11960	GAACCACCTTGCAGATTTGTTATAG
	229E-For12789	GCATGAGGGCAGAACCATTTA
	229E-For13635	GCCTGCCTTGGTGGATAAA
	229E-For14371	CGTATGTTGTCGGCTATGATCT
	229E-For15277	GCTGCTGGTCTTTGTGTAGTA
	229E-For15995	CAATGGCAAACCAGGAGAAATA
	229E-For16796	GGAGCAAGGCTGTGTTTATCT
	229E-For17590	GCTGGCTCATCTGATGTACTG
Fragment 4	229E-For17992	CGCACTGTGCAGAGTCATATT
	229E-For18811	GGTTTAACACCACCTCTCAGTAT
	229E-Rev19020	TCACAGCAGTAGCAGAGAATAAC
	229E-For19607	GGAGATGGATGCTATGGTGTAAAG
	229E-For20359	TGGCGTAACTCCACTGTTATG
	229E-For21258	GTACTGTTGCGTTAGCTTCTTATG
	229E-For22032	AGGGCACCATCTACTCTATCA
	229E-For22846	AGGTCTAACATCAGCCGTTTC
	229E-For23498	CGACAACCTAATCTTGCTCTTTAC
Fragment 5	229E-For23936	TCAGTCGTGCTCATCTTTGTG
	229E-For24698	CACCCAGCTGTTGGAATAGT
	229E-For25507	CGTGCCGAGCACTACTATAA
	229E-For26311	GTACTCCTAAGCCTTCTCGTAATC
	229E-Rev27281	TTTGTGTATCCATATCGAAACCGT

RT-qPCR for HCoV-229E

For	TTAGAGAGCGTGTTGAAGGTG
Rev	GTTCTGAATTCTTGCGCCTAAC
Probe	FAM-TCTGGGTTG/Zen/CTGTTGATGGTGCTA-3IABkFQ

RT-qPCR for SARS-CoV-2[1]

For	TTACAAACATTGGCCGCAAA
Rev	GCGCGACATTCCGAAGAA
Probe	FAM-ACAATTTGC/Zen/CCCCAGCGCTTCAG-3IABkFQ

[1]Lu X, Wang L, Sakthivel SK, Whitaker B, Murray J, Kamili S, et al. US CDC real-time reverse transcription PCR panel for detection of Severe Acute Respiratory Syndrome Coronavirus 2. Emerg Infect Dis. 2020;26(8):1654-65. doi: 10.3201/eid2608.201246.

qPCR for innate immune factors

hACTB_for	CACCATTGGCAATGAGCGGTTC
hACTB_rev	AGGTCTTTGCGGATGTCCACGT
hHPRT_for	CATTATGCTGAGGATTTGGAAAGG
hHPRT_rev	CTTGAGCACACAGAGGGCTACA
hRIGI_for	CACCTCAGTTGCTGATGAAGGC
hRIGI_rev	GTCAGAAGGAAGCACTTGCTACC
hMDA5_for	GCTGAAGTAGGAGTCAAAGCCC
hMDA5_rev	CCACTGTGGTAGCGATAAGCAG
hMAVS_for	ATGGTGCTCACCAAGGTGTCTG
hMAVS_rev	TCTCAGAGCTGCTGTCTAGCCA
hIRF3_for	TCTGCCCTCAACCGCAAAGAAG
hIRF3_rev	TACTGCCTCCACCATTGGTGTC
hIRF7_for	CCACGCTATACCATCTACCTGG
hIRF7_rev	GCTGCTATCCAGGGAAGACACA
hIFNB_for	CTTGGATTCCTACAAAGAAGCAGC
hIFNB_rev	TCCTCCTTCTGGAACTGCTGCA
hOAS1_for	AGGAAAGGTGCTTCCGAGGTAG
hOAS1_rev	GGACTGAGGAAGACAACCAGGT
hOAS2_for	GCTTCCGACAATCAACAGCCAAG
hOAS2_rev	CTTGACGATTTTGTGCCGCTCG
hOAS3_for	CCTGATTCTGCTGGTGAAGCAC
hOAS3_rev	TCCCAGGCAAAGATGGTGAGGA
hRNaseL_for	AAGGCTGTTCAAGAACTACACTTG
hRNaseL_rev	TGGATCTCCAGCCCACTTGATG
hPKR_for	GAAGTGGACCTCTACGCTTTGG
hPKR_rev	TGATGCCATCCCGTAGGTCTGT
