# Supplementary material for: A guanidine-based coronavirus replication inhibitor which targets the nsp15 endoribonuclease and selects for interferon-susceptible mutant viruses
Source: PLoS Pathog. 2025 Feb 11;21(2):e1012571. doi: 10.1371/journal.ppat.1012571 (PMC11856660; doi:10.1371/journal.ppat.1012571)
Supplement: S3 Appendix — (PDF) [file ppat.1012571.s003.pdf]

S3 Appendix: Protein quantification by automated western blot

To determine the intracellular protein levels of STAT1 and cleaved/uncleaved poly(ADP-ribose) polymerase 1 (PARP1), cells were lysed in RIPA lysis buffer supplemented with Halt protease inhibitor cocktail and EDTA (both from Thermo Fisher Scientific) and whole cell lysates were cleared by centrifugation. Proteins were separated by size using the 12–230 kDa Jess Separation Module (SM-W004) and bound with a primary antibody against STAT1 (Cell Signaling, catalog no. 9176, diluted 1:50) or PARP1 (Cell Signaling, catalog no. 9542, diluted 1:100). The STAT1 primary antibody was detected using the mouse detection module (DM-002, Protein Simple), while the PARP1 primary antibody was detected using the rabbit detection module (DM-001, Protein Simple). Protein separation and detection was performed according to the manufacturer's instructions by capillary electrophoresis, antibody binding and visualization of HRP conjugates. Next, the primary and secondary antibodies were removed using the Replex Module (RP001, Protein Simple) to allow sequential total protein detection. Protein signals were visualized using Compass Simple Western software, v.6.1.0 (ProteinSimple).
