# Supplementary figures and images for: A guanidine-based coronavirus replication inhibitor which targets the nsp15 endoribonuclease and selects for interferon-susceptible mutant viruses
Source: PLoS Pathog. 2025 Feb 11;21(2):e1012571. doi: 10.1371/journal.ppat.1012571 (PMC11856660; doi:10.1371/journal.ppat.1012571)

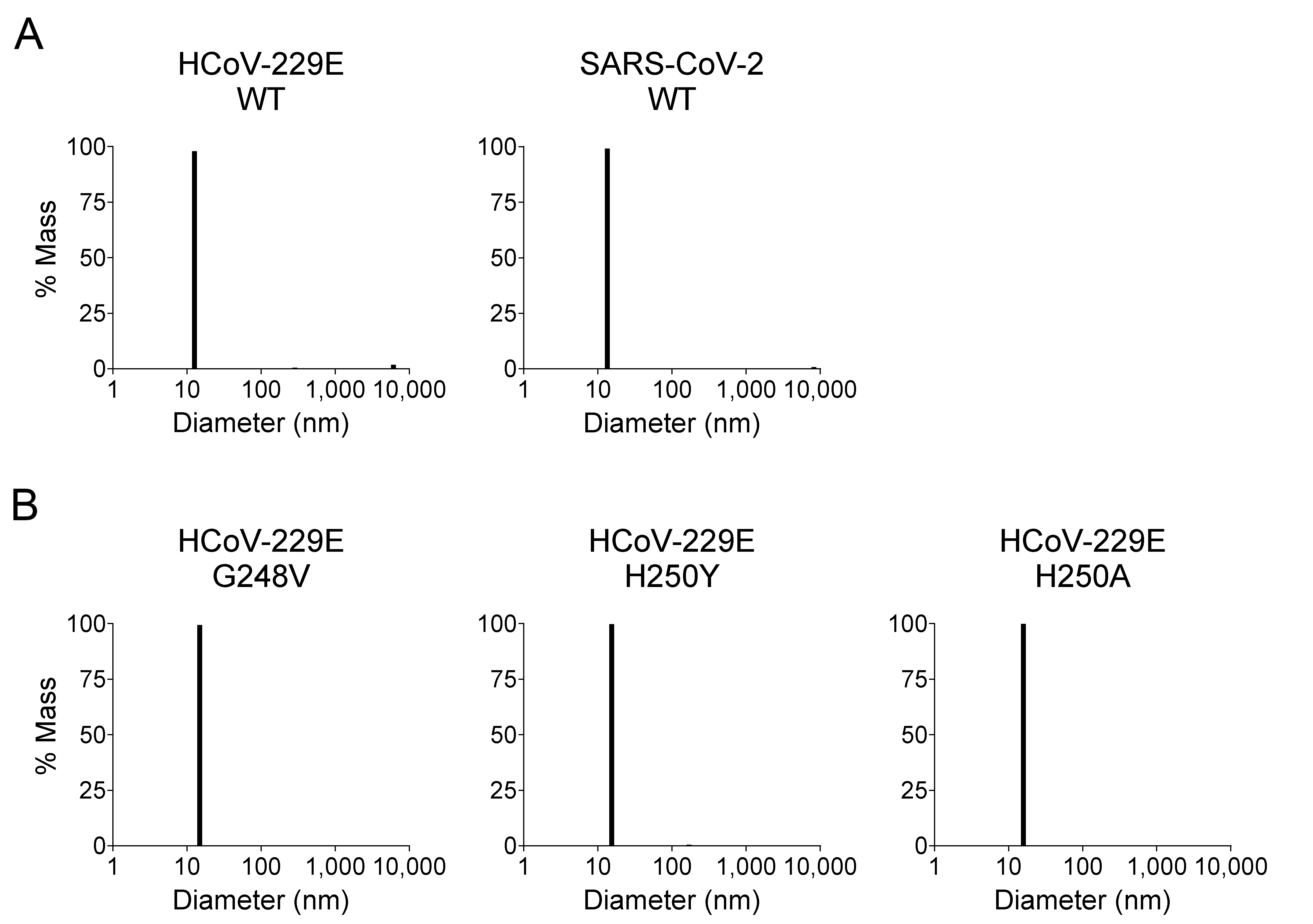

Supplement: S2 Fig — Particle size distribution of HCoV-229E and SARS-CoV-2 nsp15 as determined by dynamic light scattering (DLS). Cuvette-based DLS was performed on SEC-purified 0.5 mg/ml nsp15 solutions in storage buffer (25 mM HEPES at pH 8, 300 mM NaCl, 10% glycerol, and 1 mM tris(2-carboxyethyl)phosphine), using a DynaPro Nanostar and Dynamics software v7.10.1.21 (Wyatt Technologies). 15 µl of sample was mixed 5 µl water. The sample chamber was kept at 20 °C. The sample contained ≥ 98% particles with a diameter of ~13 nm, consistent with the size of hexameric nsp15, for both HCoV-229E (polydispersity index: 9%) and SARS-CoV-2 (polydispersity index: 11.7%). (TIF) [file ppat.1012571.s005.tif]

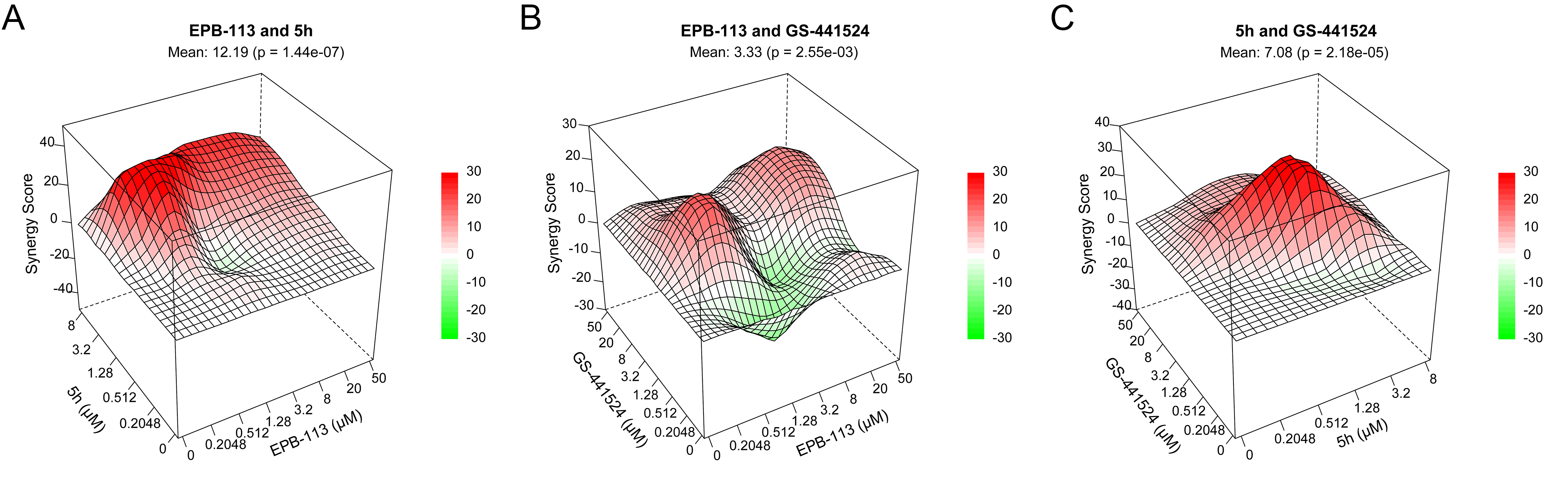

Supplement: S3 Fig — To study drug combinations, we used an alternative CPE reduction assay with HCoV-229E in HEL299 cells. An 8x8-well matrix was created, each well containing different concentrations of two compounds. Data analysis was performed with the zero-interaction potency (ZIP) model in SynergyFinder+. Mean ZIP-scores are shown on top of each graph. A ZIP-score lower than -10 indicates antagonism, between -10 and 10 corresponds with additive effects, and higher than 10 indicates synergism. The combination of EPB-113 with 5h is synergistic (A), while the combinations of EPB-113 with GS-441524 (B) and 5h with GS-441524 (C) are additive. (TIF) [file ppat.1012571.s006.tif]

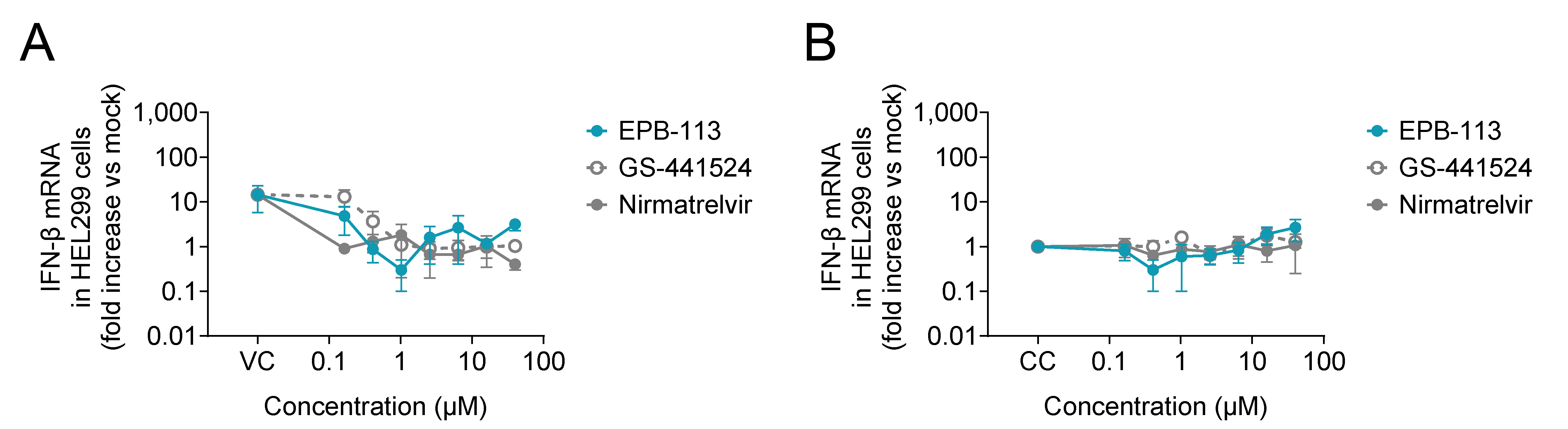

Supplement: S4 Fig — (A) EPB-113, nirmatrelvir and GS-441524 do not increase IFN-β mRNA levels in HEL299 cells when compound and HCoV-229E virus are added simultaneously. (B) The compounds do not induce IFN-β mRNA in uninfected cells. Compound exposure time: 48 h. (TIF) [file ppat.1012571.s007.tif]

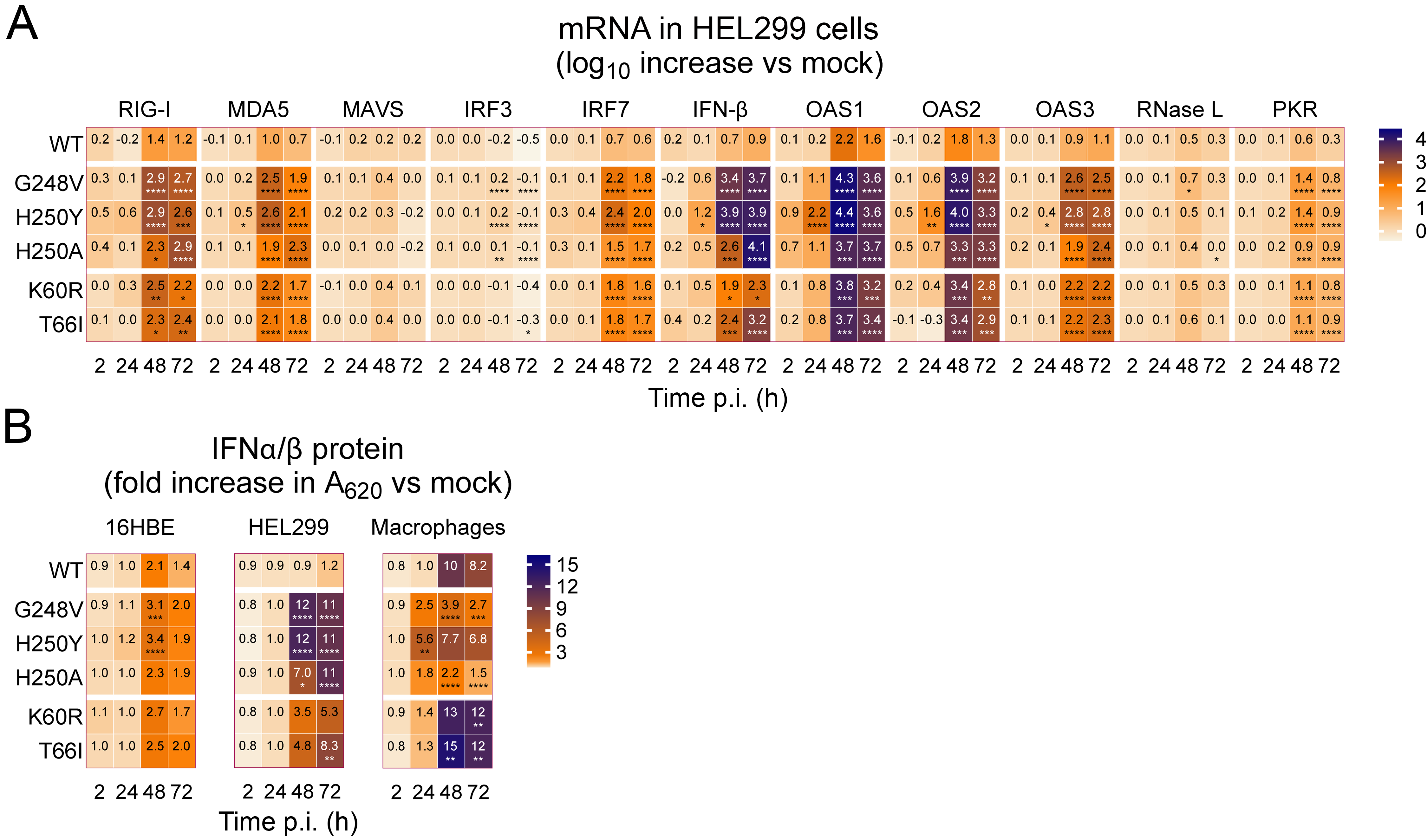

Supplement: S6 Fig — (TIF) [file ppat.1012571.s009.tif]
